# New York Odontological Society

**Published:** 1890-08

**Authors:** 


### Reports of Society Meetings.

### NEW YORK ODONTOLOGICAL SOCIETY.

The New York Odontological Society held its regular monthly meeting, Tuesday evening, April 15, 1890, in the New York Academy of Medicine, No. 12 West Thirty-first Street.

The President, Dr. J. Morgan Howe, in the chair.

### INCIDENTS OF OFFICE PRACTICE AND CASUAL COMMUNICATION.

*Dr. S. G. Perry.*—I wish to exhibit a reamer, to be held between the thumb and fingers, for the enlargement of openings in root-canals, more particularly in the back teeth. The principal peculiarity about it is that the shaft is made considerably smaller than those heretofore used. The smaller the diameter of the instrument the greater the number of revolutions it will make with a given movement of the thumb and finger, and the small diameter of the shaft of the instrument insures safety against undue strain upon the tooth and the reamer, so that there is no danger of breaking it. They can be tempered rather soft, as all steel instruments for this purpose should be, for if they have a hard temper they would break easily. These are very much shorter than the instruments of a similar pattern that have been used heretofore. When they are made short they can be carried far back even to the posterior surfaces of the wisdom teeth. They are rotated with the thumb and finger, or, being made of the size of the bur wire, they can be used in the back-action of the engine. It is very satisfactory to me to use an instrument of that sort between the thumb and fingers, because I can feel and know what I am doing, which one cannot always do when he uses the tremendous and uncertain force of the engine. They were made for me by Mr. Hodge. I have since had a quantity of them made, by Lukens and Whittington, of greater lengths, to be used for the canals of any of the teeth. The shortest ones for the back teeth are three-quarters of an inch, and the longest ones for the other teeth are three and one-half inches. The long ones can also be used in the engine, if desired, as the shaft is of the size of the bur wire.

They are intended, however, more particularly for use between the thumb and finger, and are as nearly as possible a "safety-reamer." The flanges are not deeply cut, and they "bite" very little. and are therefore not liable to break.

*Dr. Geo. S. Allan.*—Mr. President, I do not know whether it is necessary for me to call up from the table the letters of Drs. Heitzmann, Bödecker, and Abbott, which were read at the last meeting of this society, in order to reply to them. If it is not, I would like to have the privilege of making a short reply.

*The President.*—Dr. Allan is in order.

*Dr. Allan.*—It is both proper and right, having introduced the original preamble and resolution, to reply to these communications, and so I beg a little of your time in which to do so.

Dr. Bödecker's letter merits only a passing notice. First, as to the intimation that the action of the Society can only lead to unpleasantness and harm to itself, I fail to see wherein the danger lies. If any such trouble does follow, the Society will in no way be to blame for it. They only did that which seemed reasonable and right, and did it in the most courteous and gentlemanly way. If offence be taken, the fault will be elsewhere than with the Society.

It is an error to suppose the matter a personal one between Dr. Heitzmann and myself. The reasons for this I plainly pointed out in the remarks I made at the time I submitted the resolutions. That the Society agreed fully with me was made very evident by their adoption of those resolutions by a unanimous vote. It is true that I have declined to accept Dr. Heitzmann as an autocrat in matters biological, or his wild theories unsupported by facts or demonstrations. His followers, I am well aware, are willing to waive these slight formalities, but I am not, and I am happy to say I find myself in a large and very select company.

As to the letter signed by all three of these gentlemen, it is certainly a remarkable production, and I propose to show wherein this statement is true, though I doubt greatly whether it is at all necessary. The letter speaks for itself.

My amazement was great when I first read it, and for a moment or so I thought I must have been dreaming these many years, and that most unwillingly I had been maligning my neighbors. Then the truth dawned upon me, and I said, Can it be possible that they hope by so palpable an evasion as this to shut off inquiry and criticism? Either such is the truth, or it is a clear back-down.

Now, let me acknowledge in the beginning that there is just one little grain of truth in the ignorance they impute to me. It is very true, but very pardonable. I confess, then, that for the moment when writing the words "protoplasmic reticulum," I had forgotten that the gentlemen in their several writings had used the words "living matter," and, furthermore, that Dr. Heitzmann claimed the great discovery of a fibrous reticulum existing in protoplasm, and had asserted that this reticulum was the living matter of protoplasm, and that the semifluid mass in which the reticulum was embedded was dead matter.

Please note the following taken from Dr. Bödecker's writings; it seems to relate to the point at issue:

> "In 1873, Dr. C. Heitzmann (Sitzungsberichte der Kaiserl. Akad. in Wien) first discovered the minute structure of protoplasm and that of other tissues of the animal body, mainly epithelium and connective tissue. This author describes the net-like structure of the protoplasm thus: 'The nucleolus is connected with the wall of the nucleus, and this again with the granules of the protoplasm, by very fine threads, which are to be regarded as the living matter of the protoplasm, while the fluid contained within these meshes of living matter does not possess the property of life.' "

So you see that my ignorance of histological nomenclature, or the English language,—or both,—consisted in my not following literally their nomenclature and English; though, sad to relate, every other authority of note and power in histological science, the world over, uses the expressions synonymously.

Protoplasm and living matter to all others mean one and the same thing. Only with Dr. Heitzmann and his few followers have they been divorced and the one made to play a subordinate part to the other. You will all thank me, I know, for showing you my grievous mistake, and so preventing you from making yourselves liable to the same charge of ignorance. You must pay no attention to such authorities as Huxley, Spencer, Klein, Stricker, and a host of others, but stick strictly to the text as Dr. Heitzmann laid it down.

In further elucidation of my position, and to aid others in grasping the unique one held by the three gentlemen referred to, I will make a few quotations from recognized authorities. First,—though it seems almost a waste of time,—I will quote in reference to the synonymous nature of the terms "protoplasmic reticulum" and "living matter."

In the "Encyclopædia Britannica," last edition, under the heading "Protoplasm," we find as follows:

"In most of the biological articles already before the reader, whether concerned with general questions, as Biology, Anatomy, Botany, Embryology, Evolution, Histology, Morphology, Physiology, etc., or even with special groups of living beings, as Animal Kingdom, Foraminifera, Fungus, Protozoa, etc., special reference has been made to *protoplasm* as the *living matter* from which," etc. A comprehensive summary, which means that the leaders in all these various branches of knowledge use the terms as I did, and that Heitzmann's great discovery was unknown to them.

Schafer, in his "Elements of Histology," last edition, in speaking of cells, says, "These are minute portions of living substance or protoplasm."

Sedgwick and Wilson, in their treatise on biology, devote a chapter to "Living Matter or Protoplasm." It is unnecessary for me to add to this list. Practically, I have the whole literature of the subject on my side. And now as to the reticulum itself.

Klein, who almost alone of the authorities of the day connects Professor Heitzmann's name with the reticulum, makes but a brief allusion to it, and, strange to say, makes no reference to the reticulum as being the living matter of protoplasm, a most unpardonable omission, I must confess; but Klein's sins of omission are exceeded by his sins of commission, for a little further on—referring to the nucleus—he says, "This again is composed of a stroma or nucleus net-work and the interstitial substance." If this means anything it means that Klein believes the reticulum to be dead matter. Possibly Klein never knew what Heitzmann had discovered, or, what is more probable, he attached no importance to a discovery that had no better foundation for its acceptance than a dogmatic assertion of its truth. That sort of coin, as a rule, does not pass with scientists of repute.

Now, it may be a proof of ignorance on my part, but so far I have not been able to find more than one or two authorities who refer to Professor Heitzmann in any way as having made this discovery. I am willing to grant that Klein does not stand wholly alone. But most of the standard writers on the subject do refer to a reticulum that may be found in protoplasm, more easily, as a rule, in vegetable than in animal protoplasm; but nowhere else is it referred to as the essential living part of protoplasm. Dr. Heitzmann can say, in all truth, not only that he discovered this great fact, but that he stands to-day as the sole and only believer in it in this country. The exceptions are too rare to rob him of his glory.

Sedgwick and Wilson spoke of the reticulum as being difficult to discover, and as being visible only under the highest powers and with the most careful illumination. Schafer, in his "Essentials of Histology," refers to it only slightly and as *sometimes* being present. Wishing for further light, I wrote to Dr. Andrews to ask Professor Sedgwick to give me the latest German views on the subject, and the professor kindly sends me the following letter:

"I send you an extract from a poor translation (by one of my pupils) of a paper by Bütschli, in which incidentally some light is given on the structure of protoplasm by him, one of our ablest and foremost men of modern biology. I also find that Berthold, in his book on protoplasm (1886), comes to a similar view: thus (page 64), 'From the actual results of studies of the finer structure of the protoplasm and their undisputed confirmation . . . and, finally, from general theoretical considerations, it appears that the fundamental substance of the protoplasm and the different things which it contains . . . are an exceedingly complicated mixture, and that the protoplasmic mass in its entirety is to be considered as an emulsion of a more or less fluid consistency.'

"He therefore regards protoplasm as an emulsion, as Bütschli does. The finer, clearer, transparent part he does not venture to discuss at length. Heitzmann thinks this to contain a reticulum. So do the botanists Strasburger and Schmitz.

"Flemming admits a thready structure in the clearest protoplasm; but while granting that the thread *may* make a net-work, strongly doubts it.

"Berthold agrees with Flemming, and both are capital men. Both suspect also that the threads seen are post-mortem appearances.

"So far as I can judge the truth is about this: thready or net-like differentiations are to be seen; but no live man can say whether they are natural or post-mortem.

"Bütschli seems to regard them as gelatinous and (apparently) as alive. Nobody knows whether they are or not, at least *I* don't."

In conclusion, let me add that I feel certain the day is not far distant when the gentlemen whom I have referred to will feel ashamed of their action. In their weak efforts to silence me they have done more to discredit their own views with thinking men than, unsupported, I could have done in years, and I thank them heartily for the favor. Does any one doubt for a moment that if they had specimens such as they profess to have, they would not eagerly have embraced the grand opportunity offered them by this society to obtain its endorsement, and the endorsement of such a committee of experts as the preamble and resolutions called for? They were asked to co-operate with this society in the formation of a committee that would be satisfactory to them, and their reply is the claim that they themselves are the only experts available. I did not think they would take such a ridiculous position. If they are satisfied with it, I have every reason to be with my own. Evidently, and for some good reasons, they are very averse to showing their specimens.

(To be continued.)

